# A monolithic nanostructured-perovskite/silicon tandem solar cell: feasibility of light management through geometry and materials selection

**DOI:** 10.1038/s41598-020-58978-5

**Published:** 2020-02-10

**Authors:** Mahmoud H. Elshorbagy, Eduardo López-Fraguas, Fateh A. Chaudhry, José Manuel Sánchez-Pena, Ricardo Vergaz, Braulio García-Cámara

**Affiliations:** 1 GDAF-UC3M, Department Tecnología Electrónica, Universidad Carlos III de Madrid. Avda. Universidad, 30. Leganés, Madrid, Spain; 2 Physics Department, Faculty of Science, Minia University, 61519 El-Minya, Egypt

**Keywords:** Solar cells, Metamaterials, Metamaterials

## Abstract

The use of several layers of different materials, taking advantage of their complementary bandgap energies, improves the absorption in multi-junction solar cells. Unfortunately, the inherent efficiency increment of this strategy has a limitation: each interface introduces optical losses. In this paper, we study the effects of materials and geometry in the optical performance of a nanostructured hybrid perovskite – silicon tandem solar cell. Our proposed design increases the performance of both subcells by managing light towards the active layer, as well as by minimizing reflections losses in the interfaces. We sweep both refractive index and thickness of the transport layers and the dielectric spacer composing the metasurface, obtaining a range of these parameters for the proper operation of the device. Using these values, we obtain a reduction in the optical losses, in particular they are more than a 33% lower than those of a planar cell, mainly due to a reduction of the reflectivity in the device. This approach leads to an enhancement in the optical response, widens the possibilities for the manufacturers to use different materials, and allows wide geometrical tolerances.

## Introduction

The recent years have seen a boost in the research and development of perovskite solar cells, due to its remarkable increase in efficiency, ease of fabrication and performance possibilities^1,2^. In their tandem configuration with crystalline Silicon^3,4^, the way to stack all the layers is the key point. The approach with four terminals comprises a mechanical challenge, because it simply puts one subcell over the other. Each subcell is optimized in its own range, but more contact layers are necessary to extract the charges from each one, leading to important optical losses^5,6^. On the other hand, a two-terminal approach simplifies the mechanical stack, and reduces the number of contacts. However, in this case, it is mandatory that a current matching between the different subcells exists^5,6^. The photogenerated current in the top subcell must match the one of the bottom one, or there would be current losses in the extraction process. Despite these concerns, an increasing interest is arising for this configuration^7,8^.

The goal for achieving the best design of a two-terminal tandem solar cell is to reduce the optical losses as well as to improve the charge extraction, while maintaining a match between the currents photogenerated in each subcell. Focusing on the optical losses, there are two strategies to achieve this. One is the reduction of the reflection in the interfaces between layers by antireflection structures or texturing surfaces^9–12^. The other one is the increment of the light path in the active layers by resonant or diffractive nanostructures^13–18^. These strategies lie in the area of light management, which is one of the most promising ones to enhance the performance of Silicon photodetectors^19,20^ or solar cells^21^, to produce clean hydrogen^22^, to create leaf-inspired nanostructures over graphene^23^, and as in our case, for the optimization of perovskite solar cells^24^.

In a recent work, we proposed a novel design in this research line^25^. Instead of including a new nanostructure, we proposed to nanostructure the perovskite layer in a perovskite-silicon tandem solar cell. Actually, we converted the perovskite layer in a hybrid metasurface: the transport layers and a dielectric spacer form a 2D nanostructure which is filled with perovskite. This nanostructure could enhance light confinement into the perovskite volume by resonant scattering, while it also efficiently guides light towards the silicon layer underneath. Even more, the structure increases the contact surface between the transport layers (hole -HTL- and electron -ETL- transport layers) and perovskite, improving the extraction of the photogenerated charges. In that work, we performed an optimization of the geometry to achieve the maximum matched short-circuit current density of 19 mA/cm^2^, which is an increment of a ~17.2% respect to a planar cell taken as a reference^9^. Besides the proposed geometry, a further examination is required in order to obtain the best optical performance and make it compatible with the current state of the art, as well as to explore the feasibility of fabrication.

The present work shows an evaluation of the proposed nanostructure in terms of dimensions and materials, with the aim of minimizing the optical losses, mainly the reflection ones. Although this study could be performed in a 4-terminal device, the basis would be very different. Moreover, the interface layers needed in the 4-terminal configuration require a convenient index-matching to maintain the optical effects that we achieve. We study the optical losses dependences on the geometrical and optical constants of the nanostructure materials. In particular, we focus the analysis on the HTL, ETL and the dielectric spacer (DS) to find out the values ranges of geometrical parameters and refractive indices that allow a minimization of these losses. The results provide a new and wide freedom of elections in both materials and geometrical parameters to accomplish a high efficient tandem perovskite-silicon solar cell. Even more, we offer a high geometrical tolerance that allows the optimum performance of the cell.

## Setup and Method

**Figure 1:**
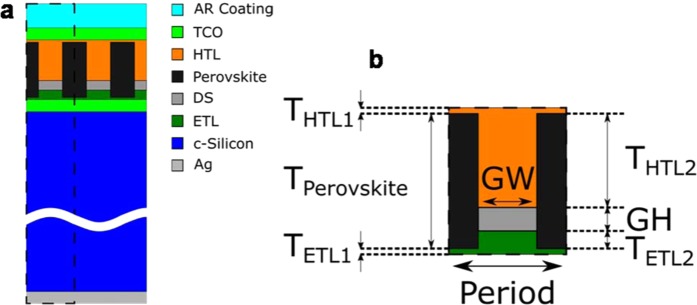
(**a)** Scheme of the proposed perovskite-silicon tandem solar cell including a hybrid metasurface. (**b)** Detail of the nanostructure, including labels of the considered geometrical parameters.

The studied structure is shown in Fig. 1. The simplified design of the solar cell comprises the following layers from bottom to top: silver contact (300 nm)/crystalline silicon (c-Si, 200 µm)/indium tin oxide (ITO, 44 nm) and a nanostructure sandwiched between the electron transport layer (ETL) and the hole transport layer (HTL), with a width regulated by a dielectric spacer (DS). Inside this structure, the perovskite fills the gaps (black in Fig. 1c). The dimensions of the dielectric spacer (width, GW, and height, GH, in Fig. 1c), regulate the perovskite volume. The ones that we used as starting points are GH = 200 nm and GW = 300 nm. A final layer of indium zinc oxide (IZO; 44 nm) and a MgF_2_ antireflection coating of 105 nm are added on top. Remaining values are: T_HTL1_ = 10 nm, T_HTL2_ = 160 nm, T_ETL2_ = 30 nm, T_ETL1_ = 10 nm. Other buffer layers, required for the electric operation of the solar cell, are not shown here because of their negligible optical effects.

This kind of structure needs particular care to be fabricated. Nevertheless, we think that it is feasible, according to the literature^26,27^. For instance, more complex arrangements are already fabricated: coral-like structures^28^, solar cells with nanostructured transport layers^29^, either the ETL^30^ or the HTL^31^, creating a kind of nanorods for the transport layers that could be embedded in the perovskite material^32^, or by blading a coating^33^.

The perovskite simulated in this work is MAPbI_3_, one of the most widely used in perovskite solar cells. The starting point of the study considers the following materials of the nanostructure: Spiro-OMeTAD for HTL, titanium dioxide (TiO_2_) for the ETL and silicon nitride (Si_3_N_4_) for the dielectric layer. The complex refractive indices of the materials are taken from references^34–41^.

In order to explore the best ranges in thicknesses and materials for each one of the layers forming the nanostructure, in which the perovskite is inserted, we calculate the optical and current losses. Thus, the methodology is based on looking for the minima of the optical absorption in the HTL, ETL and DS layers, as they are not producing effective currents, and the minimum reflection of the whole structure. In this way, we hold a GW value, like the one in Fig. 1, and seek for the value of GH and the thicknesses and refractive indices of the ETL, HTL and DS to achieve our goal. We should remark that the process is done considering each layer independently due to computational restrictions.

We calculate the device currents, both the effective photocurrent generated in the active layers (perovskite and c-Silicon) and the current losses generated in the rest of the layers, by integrating the photon flux of the incident light in a wavelength range from 300 to 1200 nm, considering an AM1.5 G (1000 W/m) spectrum^2^. We use them because the optical losses are evaluated in terms of current losses. Additionally, we also compute the short-circuit current density (J_SC_) in each layer in order to measure the device performance, as well as to preserve the current matching between the subcells.

We use the finite element method (COMSOL Multiphysics ©) to compute the optical absorption of each layer. This tool solves the Maxwell equations in each one of the small elements in which it divides the proposed system. We set periodic boundary conditions (see unit cell at Fig. 1a), and perfect matched layers at top and bottom absorbing boundaries. It is worth noting that our sweeping method of the refractive index firstly assume the ideal case of a lossless material. Once it indicates a value of *n’* providing reduced losses, we choose the realistic material closest to this value and consider its complex refractive index. This means that the final absorption and reflection simulations have been done with realistic and complex values of the refractive indices. We also compute the total reflectance and transmittance of each layer using the *S* parameters. We follow the method described in reference^25^ to validate the 2D model with normal AM1.5G illumination and considering TE (transversal electric) and TM (transversal magnetic) polarizations.

## Results

Our study implies that both the thickness of each element in our nanostructure (see Fig. 1b) and their materials can vary, in order to explore the solar cell optical operation. This may open new possibilities for the perovskite solar cell manufacturers about the material and size restrictions that they confront in the fabrication process^9^. In this section, we present a survey on the possible HTL, ETL and DS materials, as well as their thicknesses, to reduce the optical losses in the solar cell.

### Hole transport layer analysis

**Figure 2:**
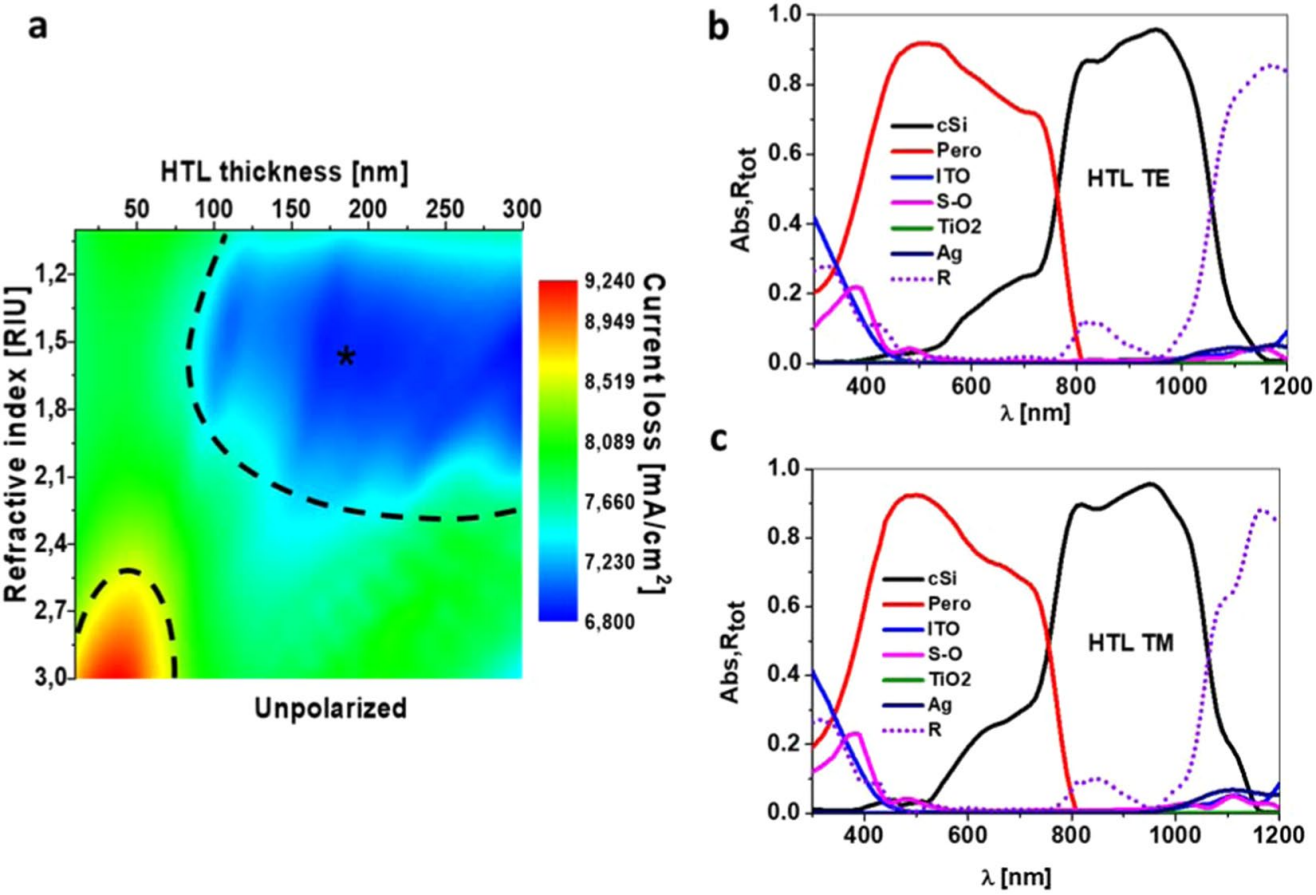
HTL analysis. (**a)** Current loss map depending of the refractive index of the HTL material used and the thickness of the HTL layer. Absorbance of every layer and total reflectance in the chosen case (*in **a**) under TE (**b**) and TM (**c**) illumination.

Figure 2a shows the results of the simulated total current losses (i.e., the sum of the losses due to reflection and parasitic absorption in the non-active layers) as a function of both the thickness T_HTL2_ and refractive index of the HTL nanostructured layer, while the rest of the parameters are fixed to the values presented above. As can be seen, the calculated losses have values within an interval from 6.8 to 9.2 mA/cm^2^. By comparing these values with the total losses of the planar case, which are 13.5 mA/cm^2^, an evident reduction of the losses using the nanostructure appears, achieving almost a reduction of a 50% in the best case. At this point, we want to highlight that there is a wide region (blue area with a dashed line limit) in which the losses are under 7.2 mA/cm^2^. This region comprises a refractive index from 1 to 2.3, and a height of the HTL layer from 100 nm to 300 nm. The first result allows the use of several materials to play the role of hole transport, such as PEDOT:PSS (refractive index of around 1.46 in the visible region, where the perovskite absorbs), or the Spiro-OMeTAD (around 1.6 in the visible). The second result allows a wide tolerance for height. This tolerance widens the possibilities for the manufacturers to obtain a good result regardless of the accuracy in the layer growth.

There is a region of high losses (bottom left red area), which comprises the refractive index interval from 2.5 to 3, and thicknesses values under 50 nm. Currently, there are no HTL materials in such interval of indices. But even at these values, the produced optical losses would be still a 32% lower than the ones of the planar cell. According to a recent review, most of the HTL materials incorporated in high efficiency perovskite cells have low refractive indices, under 2 in the visible region^42^. This means that the vast majority of materials can offer losses lower than 8 mA/cm^2^ in our model.

As an example, we show on the right side of Fig. 2(b,c) the spectral response of the structure considering SPIRO-OMeTAD (S-O, n = 1.6) as HTL, with a 190 nm height (the point marked with a star in Fig. 2a). Both the absorbance of each layer and the total reflectance (dashed line) are shown for an incident transversal electric-TE (Fig. 2b) or a transversal magnetic-TM (Fig. 2c) polarization. This simulation considers the realistic refractive index of the materials in the considered spectral range. The most remarkable result is related with the reflectance, which suffers a noticeable reduction in the whole spectral range and presents a wide band with a value close to zero. We want to remark that this HTL height selection affects the current matching, which could be retrieved by tuning the GW value without any remarkable change in the total losses. The matched short circuit current for this case is 18.85 mA/cm^2^.

### Electron transport layer analysis

**Figure 3:**
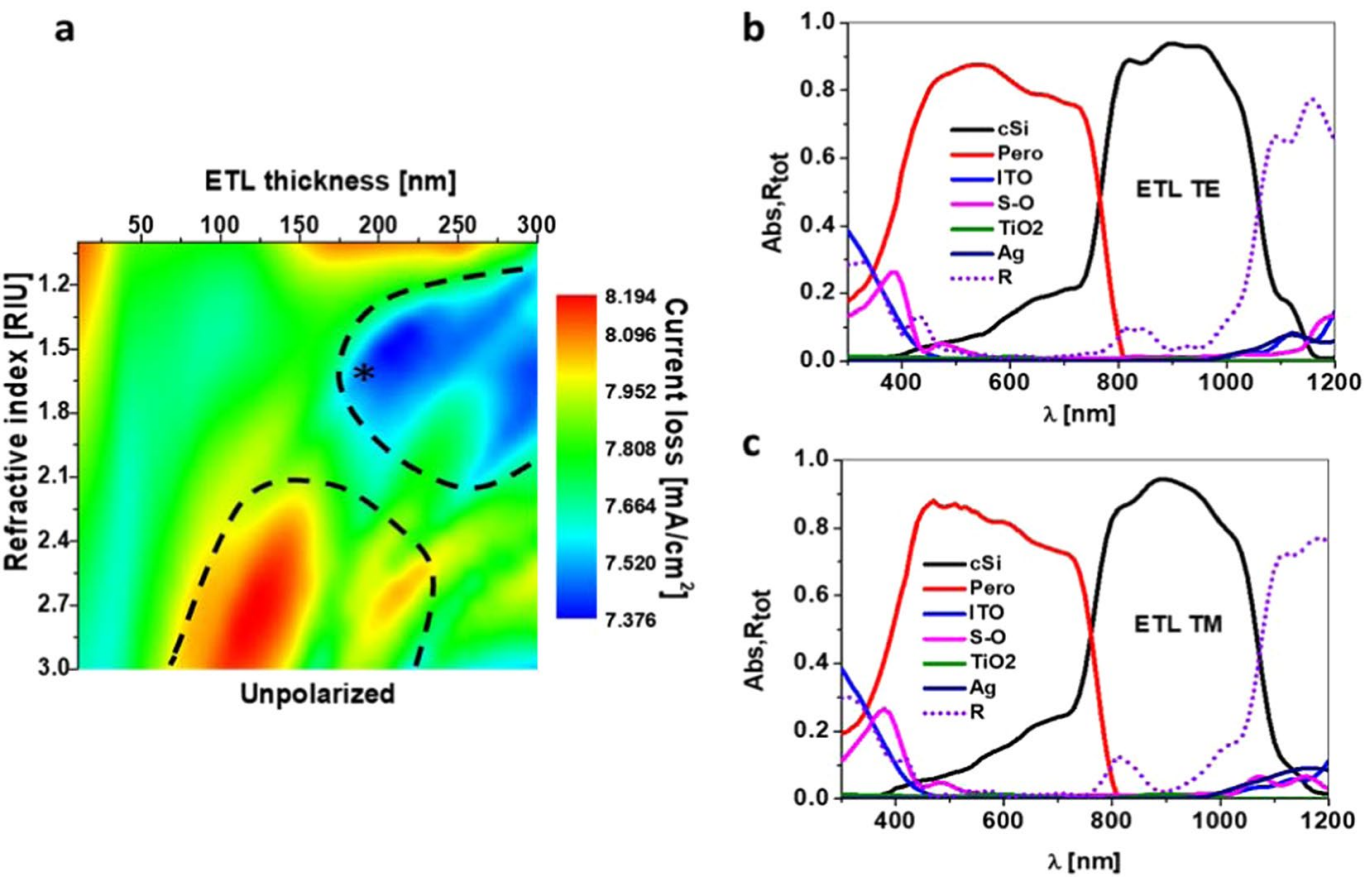
ETL analysis. (**a**) Current loss map depending of the refractive index of the ETL material and the thickness of the layer. Absorbance of every layer and total reflectance in the chosen case (*in **a**) under TE (**b**) and TM (**c**) illumination.

Following the same strategy as above, Fig. 3 shows the simulated results for the electron transport layer (ETL) analysis. As before, Fig. 3a shows a 2D map of the total losses as a function of both the thickness T_ETL2_ and the refractive index of this layer. The rest of the parameters remain as the starting point. In this case, the variation of current losses is significantly lower, ranging from 7.4 to 8.2 mA/cm^2^. The region with the minimum loss (top blue area) comprises values of the refractive index from 1.2 to 2.1, and thicknesses from 160 to 300 nm. This range allows the selection of different materials to play the role of ETL, such as SnO_2_ (around 1.85) or ZnO (around 1.6). This low-losses region is applicable to the majority of the ETL current materials, as those appearing in a recent review^43^. Although TiO_2_ (n = 2.3) is not inside this region, a low losses interval (around 7.6 mA/cm^2^) is available for this refractive index between 25 and 50 nm height.

As an example, we show in Fig. 3b,c the spectral response of the device considering ZnO playing the role of ETL, with a height of 190 nm (star in Fig. 3a). The complex refractive indices are considered, including the one of ZnO^44^. Both the reflection and parasitic absorption are reduced compared to those of the planar device. Actually, the matched short circuit current for this response is 18.9 mA/cm^2^, larger than the 16.2 mA/cm^2^ of the planar case. A GW equal to 390 nm is required to achieve the current matching.

### Dielectric spacer layer analysis

**Figure 4:**
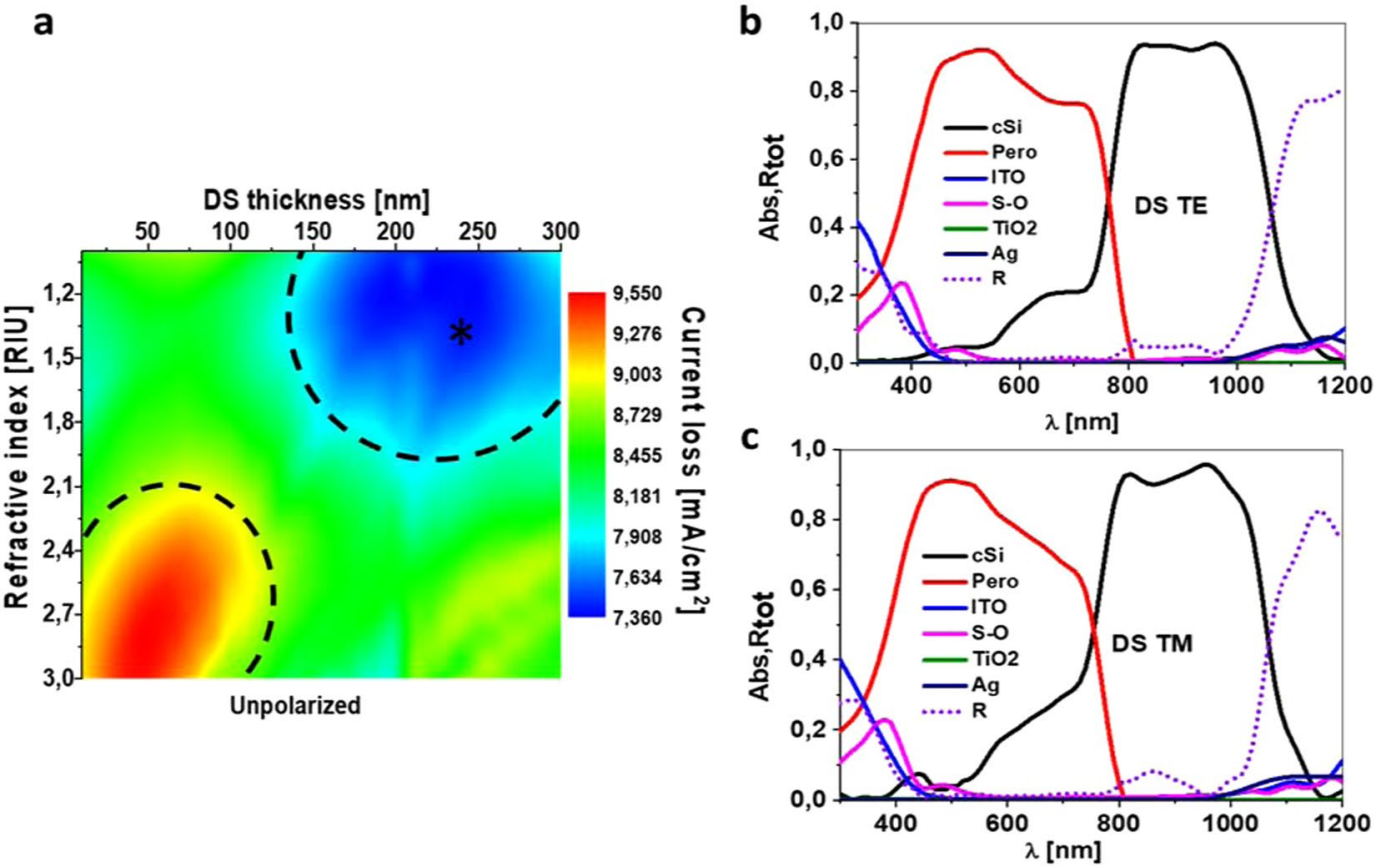
Spacer analysis. (**a**) Current loss map depending of the refractive index of the spacer material and the thickness of the layer. Absorbance of every layer and total reflectance in the chosen case (*in **a**) under TE (**b**) and TM (**c**) illumination.

The third part of the nanostructure that we study is the spacer (DS). As before, the 2D map of its current losses as a function of its refractive index and thickness is depicted in Fig. 4a. In this case, the minimum-losses region corresponds to refractive indices of 1 to 2.1, and thicknesses from 150 nm to 300 nm, with a minimum losses current of 7.4 mA/cm^2^. This refractive index range allows the use of several dielectric materials to play this role: MgF_2_ (with an index of around 1.37), SiO_2_ (1.45), PMMA (1.6), SiN_x_ (slightly over 1.8), or Si_3_N_4_ (n ≈ 2). The current losses variation into this range is noticeable, because it could reach a 7.9 mA/cm^2^ value in some areas. In this sense, MgF_2_ could be the best election, as it lies in the middle of the low loss zone and within a wide range of thicknesses, from 125 to 300 nm.

Considering the selection of MgF_2_ as dielectric spacer, with a 240 nm thickness and GW = 330 nm, the absorbance and reflectance results are shown on the right side of Fig. 4, for both incident polarizations: TE (Fig. 4b) and TM (Fig. 4c). This case provides a matched short circuit current of 19 mA/cm^2^, which is 17.2% better than the planar case (16.2 mA/cm^2^).

## Discussion

As it can be observed, there is a similar behavior in all the maps: minimum losses tend to appear at low refractive indices and high thicknesses. Low effective index of the metasurface should have a better optical matching with surrounding layers. We also guess that high thicknesses imply both the increase of the perovskite volume and a better optical guiding effect through the grating, reducing reflections^25^.

**Figure 5:**
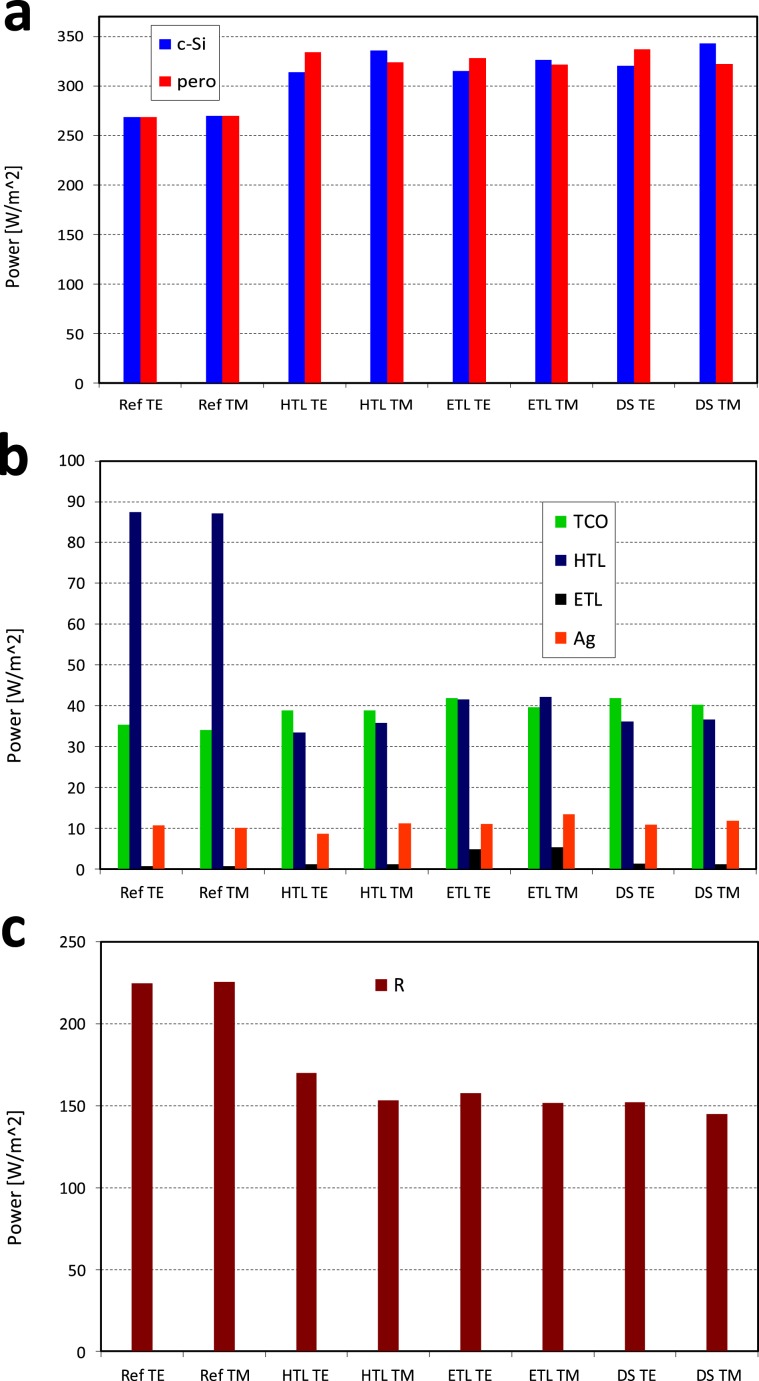
Power distribution for (**a)** the absorption at active layers, (**b)** the absorption in non-active layers (losses) and (**c)** power losses due to reflectance of the structure.

Figure 5 shows the power distributions in the layers of the planar reference and the nanostructured cases that we selected above. As before, the HTL, ETL and DS cases refer to the above selected HTL (Fig. 2b,c), ETL (Fig. 3b,c) and DS (Fig. 4b,c) cases. In particular, Fig. 5a shows the absorbed power in both the c-Si and the perovskite volumes. TE and TM polarizations are considered. While the reference case is completely polarization insensitive, the addition of the nanostructure produces certain polarization sensitivity, as expected. This means that TE/TM polarization cannot guarantee a perfect current matching in the nanostructured case. However, a non-polarized case, as that of realistic solar illumination, resembles the matching condition.

Figure 5b shows the power losses at each non-active layer for the same considered cases, while Fig. 5c shows the power losses due to the device reflectance. The total power losses of the planar reference device are 360 W/m^2^, with 225 W/m^2^ lost as reflection and the rest as parasitic losses at the transport layers and TCOs (transparent conductive oxide). The total power losses of the nanostructured case are below 250 W/m^2^, meaning a reduction of 30.5% respect to the planar case. The reason for such a reduction lays mainly in the reflection losses, as the values of Fig. 5c evidence. Actually, reflection decreases from 225 W/m^2^ in the planar case to around 150 W/m^2^ for the nanostructure cases, which is a reduction of a 33%.

On the other hand, the parasitic losses (Fig. 5b) are almost the same for all the cases, excepting for the HTL. In this case, parasitic losses are clearly reduced from about 80 W/m^2^ in the planar case to about 40 W/m^2^ (50% reduction). This is a combined effect of the selected material and the reduction of volume due to the nanostructure.

## Conclusions

This work summarizes the analysis of a nanostructured perovskite hybrid tandem solar cell. In particular, we explore the geometrical dimensions and materials of HTL, ETL and DS. This analysis provides wide ranges of both parameters for every layer. It is remarkably important from a technological point of view because it allows the use of a large number of different materials as well as offers wide fabrication tolerances. Actually, our analysis opens the possibility of using most of the available ETL and HTL materials, taking advantage of the reduction of the optical losses due to the nanostructure. Moreover, we show that there is a wide range of dimensions allowing current matching maintaining a good optical performance.

Selected cases provide a performance of the solar cell better than the planar case from an optical point of view. Additionally, we show up that the main effect over this performance is the reduction of reflectance in the different layers of the device. We obtain a 33% reduction of the total power losses due to the reflectance respect to the planar case.
